# The impact of the European Medicines Agency (EMA) and the Heads of Medicines Agencies (HMA) to address shortages of human and veterinary medicines across Europe

**DOI:** 10.3389/fpubh.2025.1673681

**Published:** 2025-10-07

**Authors:** Inga Abed, Vanessa Bennett, Brendan Cuddy, Sandra Dang, Juan García Burgos, Domenico Di Giorgio, Maria Lamas, Momir Radulovic, Nuno Simões, Rui Santos Ivo, Hugues Malonne, Monica Dias

**Affiliations:** ^1^European Medicines Agency, Department of Public and Stakeholders Engagement, Amsterdam, Netherlands; ^2^European Medicines Agency, Supply and Availability of Medicines and Devices, Regulatory Science and Innovation Task Force, Amsterdam, Netherlands; ^3^European Medicines Agency, Department of Quality and Safety, Amsterdam, Netherlands; ^4^European Medicines Agency, Supply and Availability of Medicines and Devices, Regulatory Science and Innovation Task Force, Amsterdam, Netherlands; ^5^European Medicines Agency, Public and Stakeholders Engagement Department, Amsterdam, Netherlands; ^6^Italian Medicines Agency, AIFA, Department of Inspection and Certification, Rome, Italy; ^7^Spanish Agency for Medicines and Health Products, AEMPS, Madrid, Spain; ^8^Slovenian Medicines and Medical Devices Agency, JAZMP, Ljubljana, Slovenia; ^9^National Authority of Medicines and Health Products, Infarmed, Unit for the Management of Availability and the Health System, Lisbon, Portugal; ^10^National Authority of Medicines and Health Products, Infarmed, Lisbon, Portugal; ^11^Federal Agency for Medicines and Health Products, FAMPS, Brussels, Belgium; ^12^European Medicines Agency, Supply and Availability of Medicines and Devices, Regulatory Science and Innovation Task Force, Amsterdam, Netherlands

**Keywords:** shortages, European Union, European medicines agency, medicine shortages, shortages management, HMA EMA taskforce on availability of authorised medicines for human and veterinary use

## Abstract

Shortages of medicines are a global public health challenge with a significant impact on patient care. While the issue is at the top of the agenda of EU policymakers, regulators and healthcare providers, shortages are a complex problem with many contributing factors requiring multifaceted solutions. This article is a reflection of the work EMA and network of EU regulatory agencies for medicines carried out since 2012, in particular its taskforce, set up in 2016 to provide strategic and structural solutions to tackle shortages in the EU. Since its inception, the taskforce played a key role in spearheading activities related to medicines’ shortages. Members of the taskforce supported important initiatives from leading the work on critical medicines to laying the foundation for Regulation (EU) 2022/123, which reinforces EMA’s role in crisis preparedness and management of medicinal products and medical devices. On 18 December 2024 the taskforce reached the end of its mandate. Its work is now integrated into EMA and HMA’s core activities. Future activities such as the implementation of shortage prevention plans will be further defined by measures in the revised pharmaceutical legislation, and the proposed Critical Medicines Act.

## Introduction/setting the scene

Shortages of medicines are a global issue and a critical public health challenge that is at the top of the agenda of EU policymakers, regulators and healthcare providers. The World Health Organization (WHO) and various health authorities worldwide have acknowledged the growing problem of medicine shortages, which have been affecting high-income, middle-income, and low-income countries. Shortages can affect any medicine (prescription and non-prescription) with a significant impact on patient care. Shortages can lead to medicine rationing and delay of critical treatments and can require patients to use alternatives which may be less efficacious or may increase the risk of medication errors due to unfamiliarity with the new regimen. In some cases, an absence of alternatives can mean that patients cannot be treated, and their disease may progress or worsen.

Many factors contribute to shortages, including supply chain disruptions, due to manufacturing or quality issues, regulatory issues, natural disasters, geopolitical conflicts. Other factors leading to shortages include rising demand and economic factors or the withdrawal of a product from the market by the marketing authorization holder. Shortages are not unique to the pharmaceutical sector, they are also a concern in other industries such as the microchip industry, the construction industry, the automotive industry, and the energy trade (1). In the EU, policymakers, regulators and healthcare providers are working to develop effective strategies to prevent and manage medicines shortages, as a matter of priority. The EMA/HMA taskforce on availability of authorised medicines for human and veterinary use (TFAAM, the “taskforce”) was set up in 2016 for this purpose and has now reached the end of its mandate in facilitating the prevention, identification, management and communication of shortages. Its work is being integrated into EMA and HMA’s core activities, and we therefore take the opportunity to reflect on the work completed by the taskforce. This reflection highlights the challenges overcome through close collaboration and the strategies and regulatory approaches developed, which will shape future EU efforts in addressing medicine shortages and improving public health.

## Background

Shortages of medicines have historically been managed and mitigated at a national level in affected Member States. However, between 2009 and 2011, the nature of shortages began to shift. During this period, shortages with significant international impacts started to emerge, affecting multiple countries simultaneously. Despite increased collaboration at national level between local authorities and stakeholders’ associations a coordinated EU-wide approach was lacking and a lack of early warning and structured communications between the Member States meant that the opportunities to prevent and mitigate shortages were limited. EU Member States and EMA recognized the need for coordinated action and in 2012, EMA issued a reflection paper with a series of recommendations to provide an EU-wide coordinated response to shortages caused by manufacturing or quality issues (2). These included short- and medium-term actions to effectively co-ordinate a shortage assessment, to develop risk minimization measures to alleviate its impact on patients, and to communicate within the Network, with international partners and with healthcare professionals, patients, and the general public. The reflection paper called for industry to raise awareness of the impact of shortages and also aimed to improve business continuity planning by industry to guarantee better access for patients to essential medicines. Industry stakeholders were also requested to propose solutions to further improve continuity of supply.

Building on the reflection paper, a broader response to medicine shortages, was developed through the “EU Medicines Agencies Network Strategy” and the HMA multi-annual work plan agreed in 2016 (3). The strategy emphasized the importance of information sharing, cooperation, and transparency across the EU regulatory network for medicines.

These early publications led to the founding of the taskforce in 2016. It was initially set up to provide strategic and structural solutions to tackle shortages in the EU. The taskforce focused on the availability of authorized human and veterinary medicines. This included not only shortages but also availability issues more generally, including the scenario where medicines are authorized but not marketed (so-called “access” issues).

The taskforce was a voluntary initiative between Member States and EMA to support the EU regulatory network by coordinating the development of strategies and guidance to prevent and manage shortages of medicines. Its composition included a broad group of stakeholders to ensure a comprehensive and collaborative approach to regulatory matters: representatives from national competent authorities, EMA and the European Commission, as well as the Chairs of the medicines regulatory bodies representing Iceland, Liechtenstein and Norway (the Co-ordination Group for Mutual Recognition and Decentralized Procedures—Human (CMDh), and Veterinary (CMDv) and the Chair of the GMDP Inspectors Working Group). The taskforce’s mandate operated between 2016 and 2021, was renewed in 2022 and officially ended in 2024. The work of the taskforce led to the establishment of the EU Single Points of Contact for Shortages (SPOC) network, and the delivery of policy measures, such as the Union List of Critical Medicines and Guidance for industry on implementing Shortage Prevention Plans (SPP) and Shortage Mitigation Plans (SMP) to prepare the grounds for the proposed reform of the EU pharmaceutical legislation (4).

Since its inception, the taskforce played a key role in spearheading activities related to medicines’ shortages. Members of the taskforce supported important initiatives in the area of shortages, from leading the work on critical medicines in the EU Structural dialogue (5) to laying the foundation for Regulation (EU) 2022/123 (6), which reinforces EMA’s role in crisis preparedness and management of medicinal products and medical devices. This regulation gave EMA the responsibility for monitoring medicine shortages that might lead to a crisis situation, as well as reporting shortages of critical medicines during a crisis through its Executive Steering Group on Shortages and Safety of Medicinal Products (MSSG) and SPOC Working Party.

In particular, the policies of the taskforce laid the groundwork for harmonizing ways of working between stakeholders, including wholesalers, distributors and manufacturers, to help ensure continuity in the supply of medicines. It also was key for setting up the European Joint Action on shortages CHESSMEN (Coordination and Harmonization of the Existing Systems against Shortages of Medicines—European Network) (7).

The legacy of the taskforce is evident in the robust frameworks and collaborative networks it established. These structures have significantly enhanced the EU’s ability to respond to medicine shortages, ensuring that patients have more reliable access to essential treatments.

## Achievements

### EU-wide definition for shortages and guidance on reporting

Since its inception, the taskforce identified a European-wide definition for shortages as a key enabler to developing an EU-wide approach to managing shortages. One of the first deliverables of the taskforce was therefore a harmonized definition of a shortage. This work was completed in 2019 and reflected in guidance to marketing authorization holders on detecting and reporting medicine shortages (8). It is now also included in legislation. In addition, a harmonized template for reporting shortages across the EU was developed as part of the guidance and has already been implemented in Member states (based on a survey run in 2022, 11 out of 21 responding Member States reported using either the template as published in the guidance, or a template developed based on the guidance). Ten of the responding Member States are working on implementing the guidance.

The harmonized definition of shortages and guidance for reporting also facilitated the development of a centralized electronic platform [the European Shortages Management Platform—ESMP (9)], as set out in Regulation (EU) 2022/123, to report, monitor and manage medicine shortages during crisis situations, and for the routine reporting of shortages of centrally authorized medicines. The ESMP was launched on 29 Jan 2025.

### UK’S withdrawal from the EU

The taskforce mandate also included the mitigation of risk to EU supply of medicines arising from the UK’s withdrawal from the EU which presented a significant threat to the availability of medicines. This risk of shortages was linked to regulatory changes needed from companies in the UK to be able to continue marketing the medicines in the EU.

The taskforce therefore provided practical guidance to industry on implementing regulatory changes to minimize the disruption to medicine supply and avoid shortages following the UK’s withdrawal from the EU. According to EU law, pharmaceutical companies are required to carry out certain essential operations (e.g., batch release) within the EU/EEA to market their medicines in this area. If companies had been carrying out some of these essential operations for marketed medicines in the UK, they were required to transfer them to an EU/EEA Member State before the end of the transition period (end of 2020) to comply with EU law and be able to continue supplying the EU/EEA market with their medicines.

One of the key activities of the taskforce was the identification of medicines requiring regulatory action to remain on the EU market post-Brexit and therefore at risk of shortages. During this period, the taskforce closely monitored industry preparedness for Brexit and worked directly with concerned marketing authorization holders to facilitate the necessary actions. These actions were successful and in January 2018, a survey of companies on their Brexit preparedness plans identified 108 medicines at risk of shortage, this risk was reduced to 2 medicines by November 2019.

### The SPOC network

**Figure 1:**
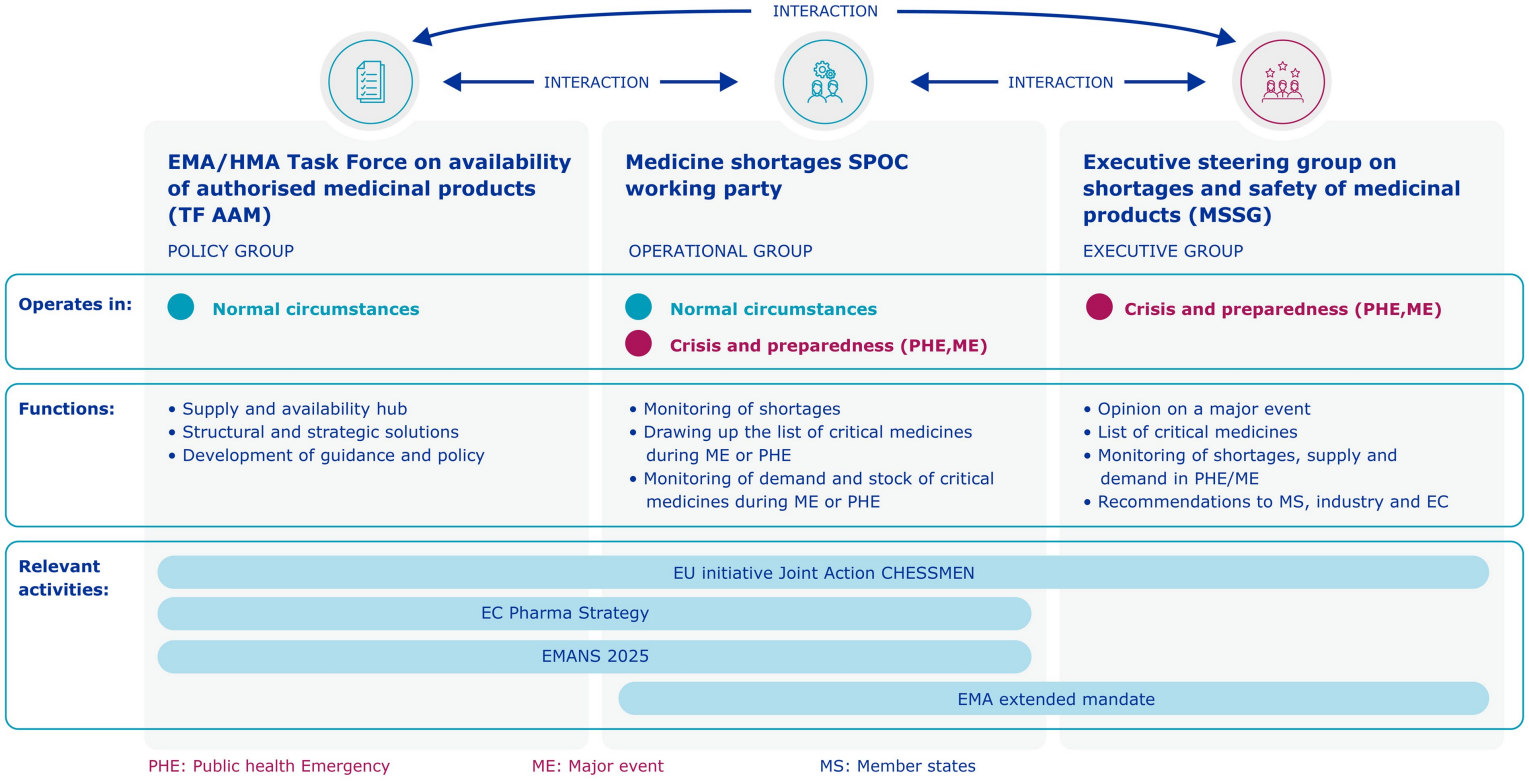
EU Cooperation on shortages after end of taskforce.

**Figure 2:**
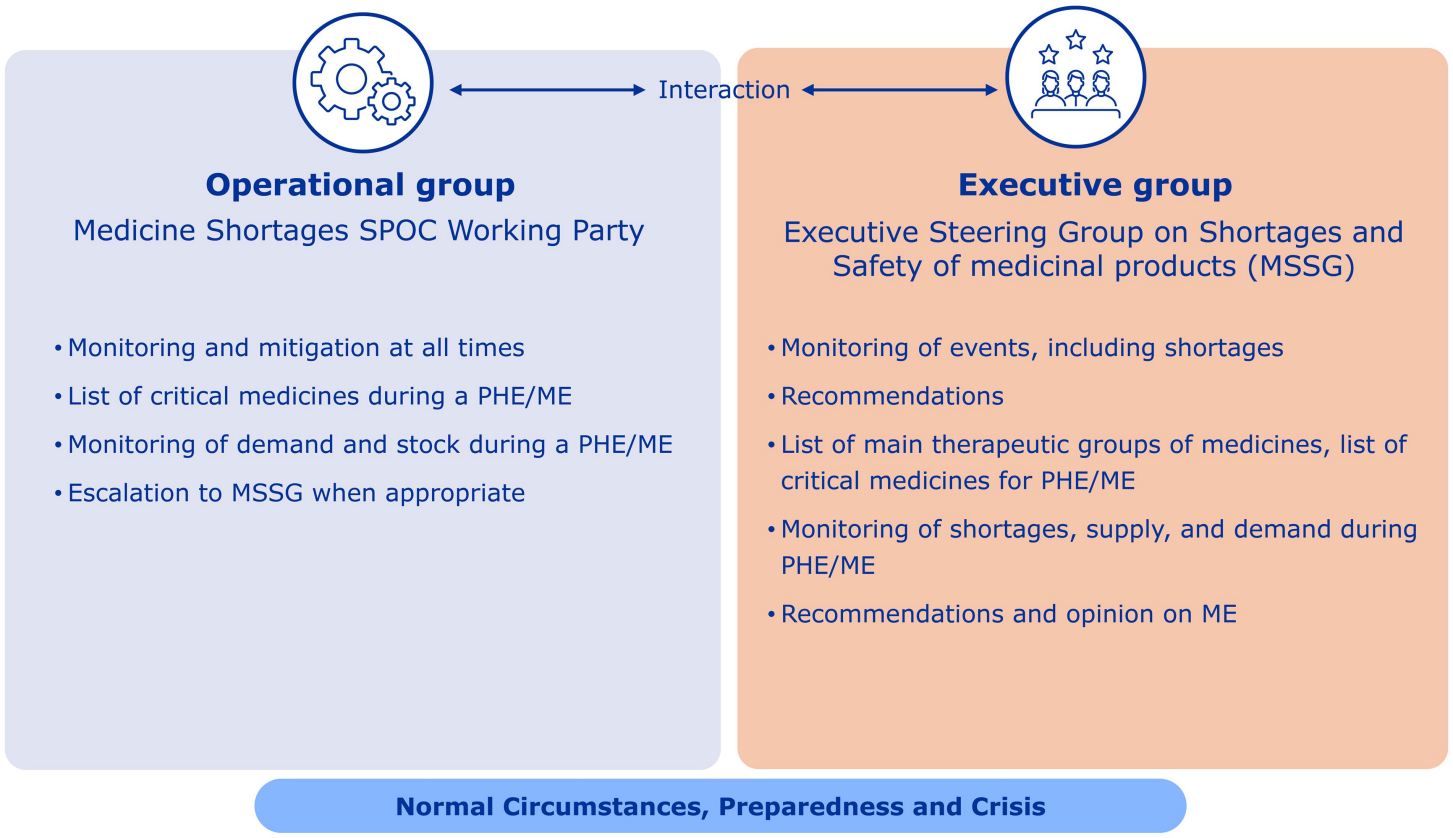
EU Cooperation on shortages with taskforce.

A key outcome of the taskforce was the establishment of the SPOC network, a network of single points of contact for medicines shortages from the national competent authorities of EU Member States. It was set up to improve information sharing between Member States, EMA and the European Commission on important human and veterinary medicine shortages and to coordinate actions to help prevent and manage shortages. The SPOC network was strengthened under EMA’s extended mandate and renamed the SPOC Working Party (SPOC WP). Today, the SPOC WP is the main actor for monitoring activities of shortages within the network and supports the MSSG to make recommendations and take EU-coordinated actions to ensure the continuity and security of supply of medicines (see Figures 1, 2). For 2024, the SPOC WP closely monitored 34 critical shortages of medicines or classes of medicines. Of these, eight were escalated to the MSSG and followed up with actions. The actions the SPOC WP and MSSG have taken include:

- Engagement with marketing authorization holders of the medicines in shortage to support them in setting up new manufacturing sites,
- Support marketing authorization holders in implementing regulatory changes such as extending shelf life of their medicine or
- implementing controlled distribution of existing supplies.
- Identifying manufacturers of alternatives, engaging with them to increase their supplies.

In four cases the newly set up voluntary solidarity mechanism (10) was used as a last resort where SPOC WP and MSSG actions were not sufficient. The mechanism allowed affected Member States to ask for support and stock of the affected medicine to other Member States (see Table 1).

**Table 1:** Critical shortages monitored at SPOC WP, escalated to MSSG and subject to solidarity mechanism in 2024.

Medicine or class of medicines affected	Critical shortages discussed at SPOC WP in 2024	Critical shortages escalated to MSSG in 2024	Critical shortage requiring solidarity mechanism
ADHD medicines	Yes	No	No
Antibiotics to treat respiratory infections	Yes	Yes	No
Atomoxetine	Yes	No	No
Cisplatin	Yes	yes	Yes
Creon and Creonipe (pancrelipase)	Yes	No	No
Cyclogyl (cyclopentolate)	Yes	No	No
Doxycycline	Yes	No	No
Ecalta (anidulafungin)	Yes	No	No
Eldesine (vindesine)	Yes	No	No
Emend (aprepitant)	Yes	No	No
Epinephrine	Yes	No	No
Fludarabine	Yes	No	No
Fluorouracil	Yes	yes	yes
GLP-1 receptor agonists	Yes	Yes	No
Insulins	Yes	Yes	No
Integrilin (eptifibatide)	Yes	No	No
IV/SC immunoglobulins	Yes	No	No
IV irrigation solutions	Yes	Yes	No
IV methotrexate	Yes	Yes	Yes
Ixiaro (Japanese encephalitis vaccine)	Yes	No	No
Menopur (menotrophin)	Yes	No	No
Mimpara (cinacalcet)	Yes	No	No
Nakom (levodopa/carbidopa)	Yes	No	No
NovoSeven (eptacog alfa)	Yes	No	No
Oncology medicines by TEVA and ACCORD	Yes	Yes	Yes
Pegasys (peginterferon alfa-2a)	Yes	No	No
Rabipur (rabies, inactivated, whole virus)	Yes	No	No
Salbutamol for inhalation	Yes	No	No
Semintra (telmisartan)	Yes	No	No
Synulox (amoxicillin/clavulanic acid)	Yes	No	No
Metalyse and Actilyse (tenecteplase and alteplase)	Yes	Yes	No
Visudyne	Yes	Yes	No
Zirabev (bevacizumab)	Yes	No	No
Zypadhera	Yes	No	No

### Guidance for the prevention of shortages

Prevention is an important aspect of shortage management and the taskforce supported proactive mechanisms to prevent shortages while trying to better anticipate and manage emerging situations. The taskforce developed dedicated guidance for all stakeholders including industry (manufacturers, wholesalers, distributors) (11) but also patients and healthcare professionals (12) on proactive mechanisms to prevent shortages. The guidance was developed together with patients and healthcare professionals whose insights played a pivotal role in shaping EMA’s approach to medicine shortages (13). Their real-world experiences provided the basis for developing the Good Practice Guidance for Patient and Healthcare Professional Organizations. The industry guidance includes recommendations on notifying shortages to national competent authorities; how to optimize the pharmaceutical quality systems to increase the resilience of supply chains, promoting fair and equitable distribution of medicines to meet the needs of patients and the importance of establishing robust shortage prevention and mitigation plans (SPPs and SMPs).

SPPs and SMPs describe strategies put in place by the marketing authorization holder to prevent or mitigate shortages. These plans can include measures such as increasing stock levels, improving supply chain management, and developing alternative sources of supply. Similar plans are also in place in other jurisdictions (e.g., the US FDA) (14). EMA is currently piloting the implementation of these plans and the first results are expected in the last quarter of 2025.

SPPs and SMPs build on recommendations for proactive assessments of risks to the manufacturing processes and supply chains by relevant industry associations [e.g., work by the Pew Charitable Trusts and International Society for Pharmaceutical Engineering (ISPE) (15)]. They are a key recommendation reflected in the communication issued by the European Commission published in October 2023 (16) which laid the ground for the revision of the pharmaceutical legislation (17) which was launched in March 2021 to address vulnerabilities in the supply chains of critical medicines, including improving security of supply and reducing dependencies. The taskforce provided input to the initial impact assessment that lead to this proposal.

### Improved communication and transparency

Effective communication and transparency are crucial for good shortage management, and communication was therefore a key pillar of taskforce activities. The taskforce was key in driving a harmonized approach to communicating on shortages. A good practice guidance for regulatory authorities for communication to the public on medicines’ availability issues was published in 2019 (and revised in 2022) (18) laying down the foundations for an improved and harmonized approach to communicating on medicine shortages in the EU/EEA. The use of shortage catalogues (publicly available systematic listings of shortages) was a key recommendation. Since the publication of the good practice guide in 2019, transparency has increased across the EU/EEA, and public catalogues of shortages are now a routine tool used by many medicines’ agencies (19). However, based on feedback from patients and healthcare professionals, further work is needed to increase awareness of the catalogues. Feedback also showed that more can be done to increase the level of details they provide and to improve the user friendliness of the catalogs (20).

The taskforce held two workshops, in 2018 and 2023, to address the issue of medicine shortages. The workshops brought together all stakeholders in the supply chain, such as national competent authorities (NCAs), the European Commission, industry, patient and healthcare professional representatives, health technology assessment bodies, payers and academia as well as veterinary medicine representatives. It provided a forum to discuss root causes and explore potential solutions and stakeholders showed a clear commitment to more solidarity, collaboration and partnership (21, 22) which will continue through the SPOC WP, MSSG, and CHESSMEN.

### Union list of critical medicines

The multifactorial nature of shortages, including unpredictable factors ranging from geopolitical tensions, natural disasters and pandemics, makes the management of shortages very challenging. A report from the Organization for Economic Cooperation and Development (OECD) found that reported shortages increased by 60% between 2017 and 2019 in European countries, the United States, and Canada (23). The COVID-19 pandemic and recent geopolitical events (such as the war in Ukraine) further compounded the problem of medicine shortages despite EMA’s activities to better monitor and coordinate actions on shortages.

A key deliverable of the taskforce was the Union list of critical medicines (24). It includes medicines that the EU identified as critical to ensure the health of its citizens and which should therefore be prioritized for EU-wide actions to strengthen their supply chain. Inclusion in the list is based on the therapeutic importance and the availability of alternatives. It includes both innovative and generic medicines for human use, spanning a wide range of therapeutic areas such as vaccines and treatments for rare diseases. While the Union list is an outcome of the taskforce, Member State authorities also provided key input together with external stakeholders and CHESSMEN. The work started in 2021 under the leadership of taskforce members through the structured dialogue initiative of the European Commission (25), an initiative informed by the Technopolis Study on medicine shortages, which provided evidence on the scope and root causes of shortages across the EU (26). The Union list was then finalized and published by the taskforce in 2023 and further updated in 2024, and will be kept up-to-date through regular revisions.

### The future for shortages management

The work of the taskforce and EMA’s extended mandate have helped to address inconsistencies and lack of harmonization in regulatory approaches at national level. The taskforce and its members have been actively involved in relevant EU initiatives related to shortages, providing a robust foundation for effective shortage management. Many of the taskforce members are today active members of the MSSG, SPOC WP and also involved in EU initiatives such as CHESSMEN.

Today, the EU regulatory network is better equipped than 15 years ago to deal with shortages and has shifted from a reactive toward a more proactive and preventative approach: shortages are a key area of focus in the European medicines regulatory network strategy (EMANS) and the SPOC WP and MSSG are key structures to ensure EU-level coordinated management and prevention of shortages. In addition, EU collaboration also continues through CHESSMEN.

Despite the taskforce’s activities to better monitor and coordinate actions on shortages, shortages persist. This persistence reflects the challenging environment and external factors, such as recent geopolitical events, that continue to exacerbate the problem of shortages. Therefore, further efforts are needed to simplify and harmonize processes, improve transparency in the supply chain, and avoid duplication of work.

Whereas the ESMP is an important step in real-time monitoring of supply data, there are opportunities for more transparency and access to critical data with the potential for artificial intelligence and machine learning to transform supply chain efficiency. This work is particularly important to address worsening external factors that pose significant challenges to shortage management.

It is important to acknowledge and build upon the progress made over the last 15 years, while recognizing the critical work that remains to be done. The taskforce has successfully established a common vision amongst Member States, fostering collaboration and cooperation through established mechanisms (SPOC WP, MSSG, and CHESSMEN). Following the closure of the taskforce in December 2024 processes have been streamlined and the SPOC WP, the MSSG’s Working Group on the Voluntary Solidarity Mechanism and Policy, and the Working Group of Communication Professionals (27), will continue the work of the taskforce with the strategic oversight of the MSSG and harmonization activities pursued by CHESSMEN. Future activities include the full implementation of shortage prevention plans and follow-up, providing a methodology for updating the Union List of critical medicines, and review of shortages root cause classification. These will be further defined and complemented by measures in the revised pharmaceutical legislation, and the proposed Critical Medicines Act (28), a regulation providing new rules and industrial policy measures to support manufacturing and improve the availability of critical medicines in the EU based on more European cooperation and coordination. Strengthening supply chain resilience and addressing sustainability challenges will be essential to ensure long-term availability and equitable access to medicines, particularly in the face of global disruptions and environmental pressures.

## In memoriam

Dedicated to the memory of Noël Wathion, former co-chair of HMA/EMA Task Force on the Availability of Authorised Medicines during its first mandate (2016-2021).
